# More than 3 years of teleconsultations: A retrospective cohort study in specialized outpatient palliative care

**DOI:** 10.1177/02692163251321717

**Published:** 2025-02-28

**Authors:** Sarah Veldeman, Tobias Martin, Johannes Wüeller, Michael Czaplik, Andreas Follmann

**Affiliations:** 1 Department of Anaesthesiology, Faculty of Medicine, RWTH Aachen University, Aachen, Germany; 2 Home Care Städteregion Aachen gGmbH, Aachen, Germany

**Keywords:** Telemedicine, remote consultation, palliative care, palliative medicine, terminal care, retrospective studies

## Abstract

**Background:**

Telemedicine in palliative care is advancing to conquer challenges like staff shortages and limited access. Though feasibility and acceptance are proven, the clinical effects of teleconsultations (a nurse on-site consulting with a remote physician) have yet to be studied. The impact on physicians’ workload or which patients it suits best, remain unclear.

**Aim:**

This study analyses the effect of teleconsultations on physician quota (number of physician-attended home visits divided by total number of home visits) and hospitalizations in specialized outpatient palliative care (SOPC) after 3 years of use in Aachen, Germany.

**Design:**

In a single-center, retrospective cohort study (September 2019–March 2023), clinical data was retrieved from a palliative care provider.

**Setting/Participants:**

1756 patients with diseases from all medical disciplines received care during the observation period. By clinicians’ choice 384 received teleconsultations, while 1372 did not.

**Results:**

833 teleconsultations were conducted. Telemedicine patients were younger (72.8 ± 12.5 years vs. non-telemedicine 74.4 ± 12.8 years, *p* = 0.011), presented more diagnoses (*p* < 0.001), while scope of symptoms and diagnoses was equivalent. Telemedicine patients had a longer duration of stay within the SOPC and more home visits. Physician quota in the telemedicine group was lower (*p* < 0.001). A matched pairs analysis (*n* = 726) showed no significant difference in hospitalizations.

**Conclusions:**

Telemedicine can reduce physician quota, alleviating personnel shortages while providing time for care-intensive patients and creating capacity for more patients. Telemedicine seems suited for multimorbid, long-term patients. A matched pairs analysis showed no difference in hospitalizations in telemedicine patients.

**What is already known about the topic?**

- Since the COVID-19 pandemic telemedicine has been on the rise in many medical disciplines and has been implemented in palliative care settings around the world.
- Telemedicine is a feasible modality for providing palliative care virtually, especially in times of contact restrictions.
- Overall acceptance for telemedical palliative care is good, although in person visits are mostly preferred.

**What this paper adds?**

- This study offers a detailed look at the clinical effects of the long-term implementation of teleconsultations (between nurse-on-site and remote physician with the possibility of point-of-care diagnostics) in the specialized outpatient palliative care setting.
- This study shows that telemedicine use in palliative care is equivalent regarding hospitalizations and can furthermore reduce physician quota hereby alleviating personnel shortages.
- The use of telemedicine is particularly beneficial for long-term patients and those with severe illnesses.

**Implications for practice, theory, or policy**

- The use of telemedicine in palliative care should be considered especially in long-term patients.
- The implementation of teleconsultations as a complementary modality of palliative care alongside regular home visits can increase time efficiency, save personnel resources, reduce physician contact times and provide more capacity for treating care-intensive patients.

## Introduction

Palliative care for terminally ill patients requires a trusting relationship, where personal contact is highly valued.^1,2^ The patients require frequent medical care ideally provided by specialized nurses and palliative care physicians.

However, home-based palliative care varies significantly across the world. The availability, frequency and quality of home visits—whether conducted by specialized nurses alone or with palliative care physicians—are determined by local resources. Access is often restricted by staff shortages, limited infrastructure, and a lack of service providers, particularly in rural areas and low-income countries, where specialized palliative care physicians are especially scarce.^3–6^ High workloads and emotional strain contribute to burnout among professionals further exacerbating the issue.^7–10^ Consequently, physician quota (number of physician-attended home visits divided by total number of home visits) varies widely across different care models and regions, and it may not be comparable or feasible everywhere.

The inadequate access to palliative care is further enhanced by increasing global demand in the light of population growth, ageing and surging multimorbidity.^11–14^ This is set against the wish of most seriously ill patients to die at home or receive care in their familiar environment as long as possible.^15,16^

As a potential solution telemedicine has entered the field of palliative medicine in the recent years, accelerated by the COVID pandemic, offering to bridge the accessibility gap, hereby saving time and personnel resources, and creating additional capacity for optimal palliative care.^17–19^

Telemedicine seems especially suited for palliative care patients,^20,21^ who need frequent reassessment, show dynamic disease progression and fluctuating symptom burden. Regular check-ups and unplanned visits, when patients deteriorate or experience sudden pain episodes, can be provided by telemedicine. Actual physician presence isn’t always necessary. Oftentimes the physicians’ expertise is required, but diagnostic measures and therapeutic interventions could be delegated to a nurse on-site.

Feasibility and acceptability of telemedical palliative care have been proven in many studies.^22–24^

But telemedicine can be provided in various forms, with videoconferencing being the most common: patients use a device (phone, tablet or computer) to speak directly to their healthcare professional. Most studies therefore examine the concept of videoconferencing.^24–27^

However, videoconferencing has limitations, as some patients, particularly the elderly or those with language barriers, may struggle with the technology. Additionally, both patients and healthcare providers express concerns that the lack of physical presence may hinder thorough health assessments, impact the personal relationship and affect the overall quality of care.^28–30^

The concept of a teleconsultation is lesser known but offers a promising alternative: a specialized nurse is on-site with the patient and consults with the remote palliative care physician.

Equipped with diagnostic tools, the nurse can facilitate a comprehensive evaluation under physician guidance, enabling expert decision-making and delegation of therapeutic measures as needed. This model of teleconsultation not only retains the personal contact and emotional support provided by the on-site nurse but also minimizes technological barriers, making telemedicine more accessible for elderly or severely ill patients, regardless of their physical fitness, social status or language barriers.

Teleconsultations haven’t been studied in the context of the specialized outpatient palliative care setting. However, the results from its implementation in nursing homes and in general practitioners’ offices were positive.^31,32^

### Objective

To assess the effect of long-term telemedical adoption in a specialized outpatient palliative care service on physician quota, home visits and the hospitalization rate of patients, this retrospective study compares data from patients who received teleconsultations with those who did not. We hypothesize that the long-term adoption of teleconsultations in palliative care reduces the physician quota without negatively affecting hospitalization rate and that the home visit rate remains unaffected.

## Methods

### Study design

This study was designed as a monocentric, retrospective longitudinal cohort study comprising a matched pairs analysis. Due to the retrospective design and anonymization of data, patient consent was not required. The study was approved by the Ethics Committee of the University Hospital RWTH Aachen (EK-23-310, February 5, 2024) and follows STROBE guidelines for cohort studies.^33^

### Setting

Over 3.5 years (September 1, 2019, to March 31, 2023), teleconsultations were performed in a specialized outpatient palliative care service (Home Care Städteregion Aachen gGmbH, Aachen, Germany) between on-site nurses and telemedical palliative care physicians. Home Care is the only specialized outpatient palliative care service in the city of Aachen (ca. 250,000 inhabitants) and the largest palliative care provider in the region. The service provides care to patients with underlying diseases from all medical disciplines.

### Care concept

Teleconsultations were conducted in addition to routine palliative care and regular home visits, which were always attended by a nurse and occasionally by a physician. Weekly team meetings comprised of nurses and physicians, identified patients for pre-planned teleconsultations. Additionally, nurses could initiate an unplanned teleconsultation for any patient during a home visit, when they needed the physicians’ expertise and medical decision.

### Study population

All patients that were listed with the service and had received at least one home visit were included in the study. Patients that were listed but did not receive any home visits or presented poor documentation quality were excluded from the study.

### Data analysis

A general analysis of all included patients (*n* = 1677) was performed, followed by a matched pairs analysis with these matching criteria: same range of duration of stay within the palliative care service (1–50, 50–100, 100–200, 200–400, >400 days) and same age ranges (20–39, 40–59, 60–79, ⩾80 years). Sex was matched wherever possible (81%). Out of 372 telemedicine patients, 349 were matched with non-telemedicine patients. 23 telemedicine patients could not be matched. These were mostly patients with an extremely long duration of stay in the palliative care service, since there were fewer long-stay patients in the non-telemedicine group.

### Telemedical system

Teleconsultations were performed using “TeleDoc Portable” system (Docs in Clouds TeleCare GmbH, Aachen, Germany), a portable case containing necessary hardware and software, including a laptop (Lenovo Thinkpad X270, Lenovo Group Limited, Hongkong, China) with TeleDoc software, a digital stethoscope (Stethoscope Littmann 3200M^®^, 3M, Saint Paul, USA) and a portable one-channel-ECG (WIWE^®^ pocket ECG, myWIWE Diagnosztika Kft, Eger, Hungary). The TeleDoc software updated from version 1.0 (2019) to version 3.10 (2023). In addition to audio and video communication, the system offers diagnostic capabilities.

Home Care gGmbH acquired the TeleDoc system in 2019 to facilitate teleconsultations as a routine part of their palliative care.

The TeleDoc system was investigated in several studies, particularly in inpatient geriatric care, regarding feasibility, user-friendliness, and acceptance.^31^ The use in palliative care has not been investigated so far.

In this study, teleconsultations were initiated by the on-site nurse through the TeleDoc software. Via audio and video communication, the palliative care physician interacted with the nurse and patient. The telemedical physician could order diagnostic measures and examinations, which were performed by the on-site nurse. The findings served as a basis for further medical decisions. If necessary, the doctor could prescribe therapeutic measures.

Data protection and secure communication were assured by end-to-end encryption.

### Data collection

All patient data was retrospectively collected from the documentation system (PalliDoc^®^ Ambulant, StatConsult Gesellschaft für klinische und Versorgungsforschung mbH, Magdeburg, Germany). The following descriptive data was systematically gathered for each included patient: age, sex, diagnoses, symptoms, number of contacts, type and timing of contact, and overall duration of stay in the palliative care service. Primary outcome parameters included physician quota and home visit rate. The hospitalization rate was a secondary outcome parameter.

### Statistical analysis

Descriptive data are presented as mean and standard deviation for normally and as median and interquartile range (IQR; Q1–Q3) for non-normally distributed continuous variables. Categorical variables are described as proportions. After normality testing via plotting and Shapiro–Wilk test, the appropriate statistical test was selected. For nominal data, *χ*^2^-test was used. For normally distributed continuous data, the unpaired *t*-test and for non-normally distributed data the Mann–Whitney *U*-test was applied. Pearson correlation analysis was employed to examine linear relationships between variables. All statistical analyses were performed using IBM SPSS Statistics 29 (SPSS Inc., Chicago, IL, USA). Statistical significance was defined as a two-sided *p* < 0.05.

## Results

### Patient Inclusion and Baseline Characteristics

**Figure 1.**
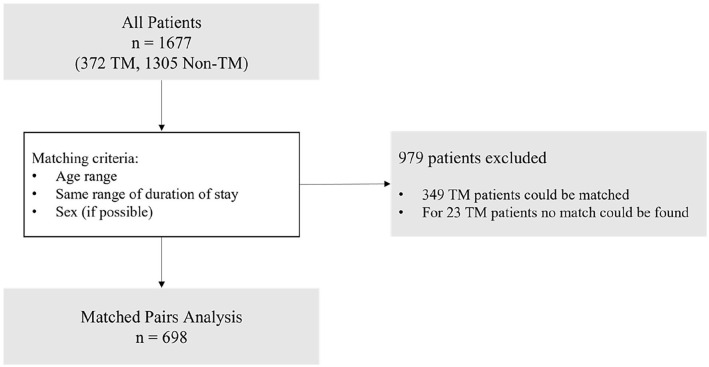
Study design. Flow chart of performed analyses. TM: telemedicine; Non-TM: non-telemedicine.

Between September 1, 2019 and March 31, 2023, 1756 patients were treated in the palliative service (Figure 1). 1677 patients were included after excluding 79 due to incomplete documentation. The mean age of entry into the palliative care service was 74.0 ± 12.7 and 864 (51.5%) patients were female.

372 (21.8%) patients received teleconsultations (telemedicine group) and 1305 (78.1%) did not (non-telemedicine group).

On average telemedicine patients were younger (72.9 ± 12.3 vs 74.3 ± 12.8) (*p* = 0.011).

In total 833 teleconsultations were performed during the study period.

### Symptoms and diagnoses

The scope of symptoms presented during patient contacts was equivalent for both groups, as well as leading diagnoses. The total number of diagnoses per patient was higher in the telemedicine group (median 10 (Q1 8–Q3 13)) compared to the non-telemedicine group (median 9 (Q1 6–Q3 12)) (*p* < 0.001); see Table 1.

**Table 1.** Overview of the Top-5-symptoms and Top-5-diagnoses during patient contacts (home visits, teleconsultations) in the general patient population and for the subgroups TM and non-TM. symptoms and diagnoses summed up for all patient contacts.

Top 5	All (*n* = 1677)		TM (*n* = 372)		Non-TM (*n* = 1305)	
	Symptoms	No.	Symptoms	No.	Symptoms	No.
1.	Weakness	13,450	Weakness	3791	Weakness	9654
2.	Pain	8368	Fatigue	2809	Pain	5937
3.	Fatigue	7194	Pain	2427	Fatigue	4380
4.	Loss of appetite	4572	Loss of appetite	1259	Loss of appetite	3308
5.	Dyspnea	4365	Dyspnea	1225	Dyspnea	3137
Top 5	All (*n* = 1737)		TM (*n* = 384)		Non-TM (*n* = 1353)	
	Diagnoses	No.	Diagnoses	No.	Diagnoses	No.
1.	Malignant neoplasm of the bronchi and lung	333	Malignant neoplasm of the bronchi and lung	74	Malignant neoplasm of the bronchi and lung	259
2.	Malignant neoplasm of the pancreas	174	Malignant neoplasm of the pancreas	39	Malignant neoplasm of the breast	100
3.	Malignant neoplasm of the breast	122	Malignant neoplasm of the breast	34	Malignant neoplasm of the pancreas	96
4.	Heart failure	109	Malignant neoplasm of the prostate	26	Heart failure	92
5.	Malignant neoplasm of the prostate	105	Chronic obstructive pulmonary disease	24	Malignant neoplasm of the prostate	79

TM: telemedicine; Non-TM: non-telemedicine.

### Duration of stay within the specialized outpatient palliative care service

**Figure 2.**
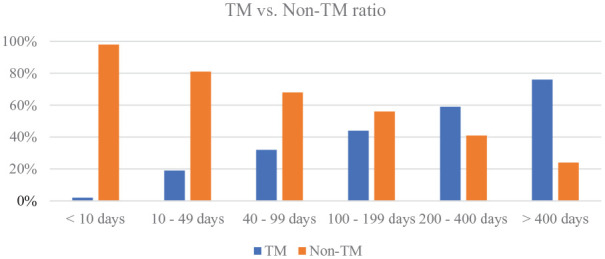
TM versus non-TM ratio. Percentage of patients within a certain duration of stay that received teleconsultations versus those that did not receive telemedical care. TM: telemedicine; Non-TM: non-telemedicine.

The overall duration of stay within the palliative care service was significantly longer in the telemedicine group (*p* < 0.001; see Table 2). The ratio of telemedicine patients versus non-telemedicine patients increases with longer duration of stay (see Figure 2).

**Table 2.** Overview of patient-specific characteristics of all patients and both TM and non-TM subgroups.

	All (*n* = 1677)	TM (*n* = 372)	Non-TM (*n* = 1305)	*p*-Value
*Demographics*				
Age—years—mean ± SD	74.0 ± 12.7	72.9 ± 12.3	74.3 ± 12.8	**0.011**
Sex—female/male—no. (%)	864 (51.5)/813 (48.5)	193 (51.9)/179 (48.1)	671 (51.4)/634 (48.6)	0.874
*Diagnoses*				
Number of diagnoses—median (Q1–Q3)	9 (6–12)	10 (8–13)	9 (6–12)	**<0.001**
*Duration of stay within the SOPC*				
Duration of stay (days)—median (Q1–Q3)	30 (9–70)	73.5 (36–146)	21 (6–52)	**<0.001**

TM: telemedicine; Non-TM: non-telemedicine; SOPC: specialized outpatient palliative care service; SD: standard deviation. Bold p-values indicate statistical significance (p < 0.05).

### Physician quota

**Figure 3.**
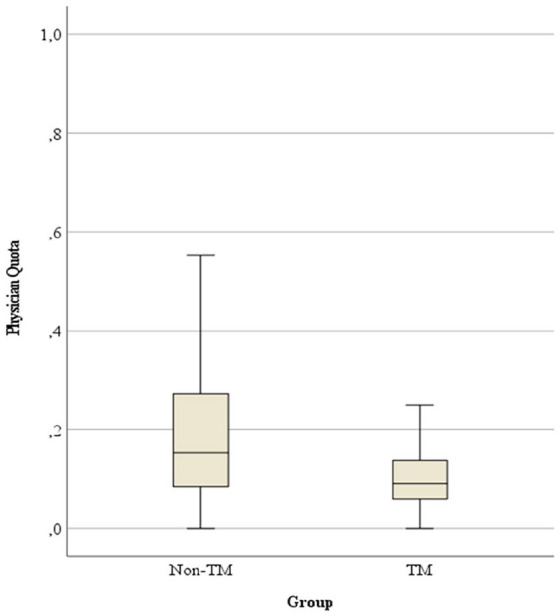
Physician quota. TM: telemedicine; Non-TM: non-telemedicine.

Physician quota (physician-attended home visits /total number of home visits) was significantly higher in the non-telemedicine group (median 0.15 (Q1 0.08–Q3 0.27)) than in the telemedicine group (median 0.09 (Q1 0.06–Q3 0.14); *p* < 0.001; see Figure 3).

### Home visits

The total number of home visits, including both physician-attended and nurse-only visits, was significantly higher in the non-telemedicine group (median 1.2 (Q1 1.0–Q3 1.7)) than in the telemedicine group (median 1.1 (Q1 0.9–Q3 1.3)) (*p* < 0.001).

### Hospitalizations

A positive correlation existed between duration of stay within the palliative service and the number of hospitalizations (*p* < 0.001). The hospitalization rate (number of hospitalizations/number of days within palliative care service) was significantly lower in the telemedicine group (median 0, min 0.0, max 0.1) than in the non-telemedicine group (median 0, min 0.0, max 0.5; *p* < 0.001).

### Matched pairs analysis

A matched pairs analysis was performed including a total of 698 patients. The physician quota was significantly lower in the telemedicine group (*p* < 0.001). The total number of home visits was higher in the telemedicine group (*p* < 0.001). Concerning hospitalizations, the matched pairs analysis showed no significant differences between the two groups (*p* = 0.594; Table 3).

**Table 3.** Overview of the performed matched pairs analysis.

Matched pairs analysis (*n* = 726)	TM (*n* = 363)	Non-TM (*n* = 363)	*p*-Value
*Physician quota*			
Physician quota—median (Q1–Q3)	0.09 (0.07–0.14)	0.12 (0.07–0.19)	**<0.001**
*Home visits*			
Number of home visits per patient—median (Q1–Q3)	77 (41–133)	64 (32–111)	**<0.001**
*Hospitalizations*			
Number of hospitalizations per patient—median (Q1–Q3)	0 (0–1)	0 (0–1)	0.594

TM: telemedicine; Non-TM: non-telemedicine. Bold p-values indicate statistical significance (p < 0.05).

## Discussion

### Main findings of the study

This study shows that implementing teleconsultations in specialized outpatient palliative care can reduce the number of physician-attended home visits (physician quota), hereby alleviating personnel shortages and providing a preventive approach to physician burn-out without increasing hospitalizations.

Telemedicine was effectively applied across the patient spectrum with no differences concerning sex or scope of symptoms and diagnoses. This is supported by similar findings of several other studies and reviews.^25–27^

Notably, the number of diagnoses per patient was higher in the telemedicine group, suggesting a higher level of multimorbidity. It’s possible that these more complex cases were intentionally selected for telemedicine to enable closer, physician-monitored care, as teleconsultations allow frequent, physician-backed check-ins.

The data also suggests telemedicine was more common in younger patients, which may correlate with longer survival rates, thus increasing opportunities for telemedical care. Telemedicine also seemed more suited for long-term patients with established trust in the care team. Such patients, who were well known to the staff, could benefit from teleconsultations for regular check-ins or additional supervision from palliative physicians. Furthermore, the longer stays of telemedicine patients may indicate that telemedicine was a reliable monitoring method during stable phases of illness. Short term patients usually require personal home visits to build trust and accurately assess patient status, especially during initial assessments, which typically telemedicine can’t replace.^34^ Additionally, patients that stay within the palliative care service for a very short time are usually already in the final stage of their disease and were specifically referred to the service to enable a dignified death at home. In these cases, physicians might favor personal home visits to comfort patients and family and guide them through the distressing final days.

Concerning hospitalizations, the general analysis showed a higher rate in the non-telemedicine group, though the matched pairs analysis revealed no significant difference. This suggests that telemedicine provides a comparable quality of care and does not lead to more hospitalizations, aligning with studies, which found no difference regarding hospitalizations,^35^ while other studies reported reduced hospitalizations and reduced emergency service interventions.^36^

The number of home visits was significantly higher in the telemedicine group. This suggests that teleconsultations served as a supplement rather than a replacement for in-person visits, offering ad hoc support and quick physician input when needed. This also underscores that telemedicine was likely prioritized for multimorbid patients requiring frequent checks. However, despite more home visits, the physician quota was lower for the telemedicine group, meaning telemedicine helped reduce the demand for physicians to be physically present during every visit.

### What this study adds

These results highlight the potential of telemedicine to save time and physician resources by reducing physician attended home visits. Considering personnel shortages and global lack of access to palliative care, it becomes clear that telemedicine can bridge the gap between increasing demand for palliative care and insufficient care options. By implementing telemedicine in the specialized outpatient palliative care setting, the time frame for home visits of especially care intensive patients can be increased while creating capacity for treating more patients overall.

### Strengths and weaknesses of the study

There are several limitations to this study. The retrospective study design and unequal sample sizes present limitations, with fewer telemedicine patients than non-telemedicine patients. This was addressed through a matched pairs analysis with balanced sample sizes. Another limitation is the monocentric nature of the study, as data from only one German palliative care service limits generalizability. Additionally, the study period overlaps with the COVID-19 pandemic, complicating the assessment of its specific impact on findings. Varied documentation quality and lack of specific teleconsultation outcome data also represent limitations. Moreover, the study did not analyze why certain patients received teleconsultations while others did not, nor did it consider the perspectives of specialized nurses, whose input is essential in palliative care settings.

Despite these limitations, a significant strength is the study’s 3.5-year duration, allowing for robust analysis with a considerable sample size of 1677 patients, including 372 telemedicine patients. This substantial dataset supports the reliability of the findings.

Further research is needed to confirm our findings. Future studies should be prospective and include patients ideally from different palliative care providers. Including specialized nurses’ perspectives would also provide a comprehensive view of telemedicine’s impact on palliative care quality.

## Conclusion

Long-term implementation of teleconsultations can reduce physician quota, hereby providing more time for home visits of especially care intensive patients while creating capacity to treat more patients overall. The use of telemedicine appears particularly beneficial for long-term patients and those with severe illnesses. A matched pairs analysis showed no significant difference regarding the number of hospitalizations, indicating an equivalent quality of care in telemedically treated patients. Further research and prospective studies could support these findings.
